# Antitumor and Adjuvant Activity of λ-carrageenan by Stimulating Immune Response in Cancer Immunotherapy

**DOI:** 10.1038/srep11062

**Published:** 2015-06-22

**Authors:** Min Luo, Bin Shao, Wen Nie, Xia-Wei Wei, Yu-Li Li, Bi-Lan Wang, Zhi-Yao He, Xiao Liang, Ting-Hong Ye, Yu-Quan Wei

**Affiliations:** 1 State Key Laboratory of Biotherapy and Laboratory for Aging Research, West China Hospital, Sichuan University, and Collaborative Innovation Center for Biotherapy, Chengdu, Sichuan 610041, PR China; 2 Department of medical oncology, Cancer Center, West China Hospital, West China Medical School, Sichuan University, Chengdu, Sichuan 610041, PR China

## Abstract

λ-Carrageenan is a seaweed polysaccharide which has been generally used as proinflammatory agent in the basic research, however, how the immunomodulating activity of λ-carrageenan affects tumor microenvironment remains unknown. In this study, we found that intratumoral injection of λ-carrageenan could inhibit tumor growth in B16-F10 and 4T1 bearing mice and enhance tumor immune response by increasing the number of tumor-infiltrating M1 macrophages, DCs and more activated CD4^+^CD8^+^ T lymphocytes in spleen. In addition, λ-carrageenan could enhance the secretion of IL17A in spleen and significantly increase the level of TNF-α in tumor, most of which was secreted by infiltrating macrophages. Moreover, λ-carrageenan exhibited an efficient adjuvant effect in OVA-based preventative and therapeutic vaccine for cancer treatment, which significantly enhanced the production of anti-OVA antibody. The toxicity analysis suggested that λ-carrageenan was with a good safety profile. Thus, λ-carrageenan might be used both as a potent antitumor agent and an efficient adjuvant in cancer immunotherapy.

Most patients with cancer receive surgery, radiation therapy, chemotherapy or combination of these treatments. However the diminishment of cancer is hard to achieve due to the restricted region for surgical procedure, development of drug-resistance, occurrence of harmful side effects and *etc*^1^,^2^. For several decades, efforts have been devoted in seeking and developing safer and more effective therapy for cancer treatment. Cancer immunotherapy has arisen from the development of oncology and immunology and gained much attention^3^,^4^. The concept of cancer immunotherapy is based on the activation of immune system to attack the tumor cells by using cancer antigens as targets and which could be achieved by using monoclonal antibodies, adoptive cell transfer and *in vivo* cancer vaccines^5^. When it comes to the cancer vaccine, the addition of adjuvant is welcomed due to which might potentiate the immune response to an antigen, such as the protein antigens, and/or modulate it towards the desired immune response^6^,^7^. The development and evaluation of appropriate adjuvant is considered as an important issue in the field of cancer immunotherapy^8^,^9^.

Immunologic adjuvants mainly include inorganic compounds^10^,^11^, bacterial products^12^, cytokines^13^,^14^, *etc.* There are also other potential molecules still under evaluation which could be possibly used as adjuvants for their immunomodulatory characteristics. Seaweed polysaccharides are reported as the immune regulators which could activate the immune cells and improve the body’s immune function^15^,^16^. Some of the seaweed polysaccharides are investigated in biomedical research and have been known for biological activities such as antitumor, antivirus, antihyperlipidemia and anticoagulant acvtivities^17^,^18^. Polysaccharides functioned as adjuvants in cancer immunotherapy appeared to have promising effects for the targeted immunity stimulation^19^,^20^. Among them, the sulfated modified polysaccharide has gained much attention^15^.

Carrageenans are mucopolysaccharides from the cell walls of the marine red algae which are anionic linear polymers composed of 1,3α -1,4β-galactans^21^. According to the different number and position of the ester sulfate groups on the repeating galactose units, they can be divided into κ-,ι-,λ- three groups^21^,^22^. Non-gelling λ-carrageenan, which has three sulfating sites every disaccharides unit, is used to induce inflammation and inflammatory pain in the rodent hindpaw or air pouch model^23^,^24^. Recently, the anticancer effect of carrageenan was revealed and Zhou *et al.* has reported that κ- or λ-carrageenan showed antitumor and immunomodulating activities in S180 and H22 transplanted mice^25^,^26^. However, in most of the tumors and immunology experiments, λ-carrageenan was administered systematically, such as administered orally or intraperitoneally^25^,^26^. It has not been studied whether or not the local intratumoral injection of λ-carrageenan has an antitumor and immunomodulatory effect. Also, few studies have used this polysaccharide in vaccines for cancer immunotherapy^27^.

In this study, we investigated how the intratumoral injection of λ-carrageenan affects the tumor growth and regulates tumor microenvironment in murine melanoma model and mammary cancer model. Also, the adjuvant effect of λ-carrageenan was studied by using antigen OVA and E.G7-OVA tumor as the model. The antitumor effect of λ-carrageenan as an adjuvant was evaluated and the antitumor mechanisms of λ-carrageenan were studied.

## Results

### λ-Carrageenan inhibits tumor growth in B16-F10 and 4T1 bearing mice

**Figure 1:**
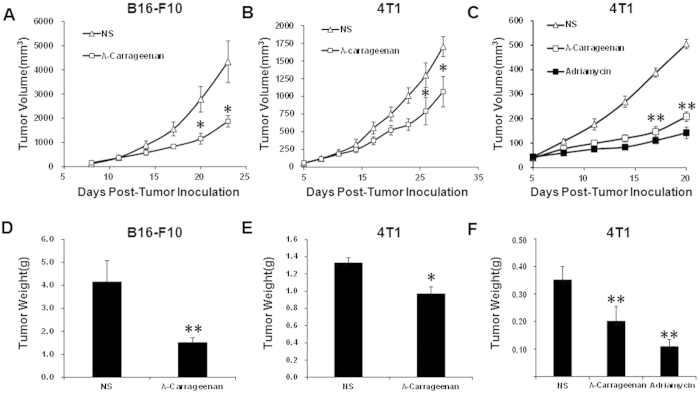
The antitumor effect of λ-carrageenan via intratumoral injection in mice. λ-Carrageenan were injected at a dose of 50 mg/kg every two days. Tumor volumes and tumor weights were recorded. NS is for normal saline group. (**A**, **D**) Tumor growth of B16-F10 cells in C57BL/6 mice with or without λ-carrageenan treatment. B16-F10 cells were subcutaneously inoculated. (**B**, **E**) Tumor growth of 4T1 cells in BALB/c mice with or without λ-carrageenan treatment. 4T1 cells were subcutaneously inoculated. (**C**, **F**) Tumor growth of 4T1 cells in BALB/c mice with or without λ-carrageenan treatment. 4T1 cells were inoculated into mice fat pad of the mammary gland. Adriamycin was used as the positive control via intraperitoneal injection at a dose of 5 mg/kg every three days. For determination of tumor weight, tumors were harvested two days after final administration. Results are presented as average ± SEM (*n* = 6, ANOVA, *p < 0.05).

To investigate how intratumoral injection of λ-carrageenan affects tumor microenvironment, we have selected melanoma B16-F10 and mammary cancer 4T1 as the models. B16-F10 cells (5 × 10^5^ cell/mice) were injected subcutaneously in mice (Fig. 1A) and 4T1 cells (1 × 10^6^ cell/mice) were injected subcutaneously into the right dorsal flank (Fig. 1B) or injected subcutaneously into mice fat pad of the mammary gland (Fig. 1C) to establish the tumor model. The administration started after the tumors reached the average volume of 30–40 mm^3^. λ-Carrageenan was injected every two days intratumoraly at a dose of 50 mg/kg and the tumor volume was recorded. The intratumoral injection of λ-carrageenan led to a significant reduction in tumor volumes while compared with normal saline groups in three tumor models (Fig. 1). We noticed that the antitumor effect of λ-carrageenan was comparable to that of Adriamycin in the 4T1 model (Fig. 1C). As calculated by the formula in the Methods (1), the inhibition rate of tumor was 56.8%, 39% and 42.7% in B16-F10 and 4T1 models respectively. Also, we noticed that λ-carrageenan had more potent antitumor effect in B16-F10 tumor model, thus, we chose B16-F10 tumor as the model to further study how λ-carrageenan affected the microenvironment in tumors.

### Effects of λ-carrageenan on cell viability of B16-F10 and 4T1 *in vitro*

**Figure 2:**
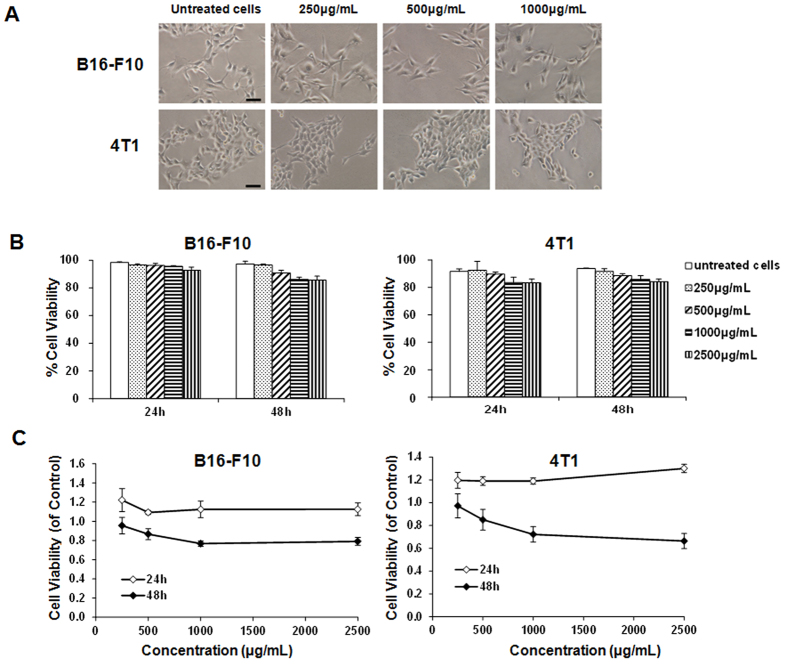
The cytotoxicity of λ-carrageenan *in vitro*. (**A**) Cell morphologies of B16-F10 and 4T1 after the treatment of various concentrations of λ-carrageenan for 24 h. Scale bar, 20 μm. (**B**) Cell viabilities after the treatment of λ-carrageenan determined by trypan blue exclusion assay. (**C**) Inhibition of cell proliferation by λ-carrageenan as detected by MTT assay. Results are presented as average ± SD (*n* = 6).

As the intratumoral injection of λ-carrageenan had a significant antitumor effect in mice, we further investigated that whether the tumor growth inhibition was induced by the direct cytotoxicity of λ-carrageenan. After the incubation of tumor cells with λ-carrageenan *in vitro*, the cell morphologies were recorded by microscopy and the cell viability was assessed by trypan blue exclusion test. B16-F10 and 4T1 cells were incubated with λ-carrageenan at a concentration of 0.25–1.0 mg/ml for 24 h and the cell morphologies were identified normal (Fig. 2A). Trypan blue exclusion test showed that even at the high concentration of 1.0 mg/ml, cell viabilities maintained above 80% (Fig. 2B), which indicated a low cytotoxicity of λ-carrageenan to tumor cells. Furthermore, the results of MTT cell proliferation assay had similar results (Fig. 2C). The incubation of tumor cells with λ-carrageenan showed no effect to cell proliferation after 24 h of treatment. After exposure to λ-carrageenan for 48 h, the relative cell viability decreased a little as the concentration of λ-carrageenan increased. Thus, the results suggested that λ-carrageenan had low cytotoxicity to tumor cells *in vitro*.

### Intratumoral injection of λ-carrageenan stimulates tumor immune response

**Figure 3:**
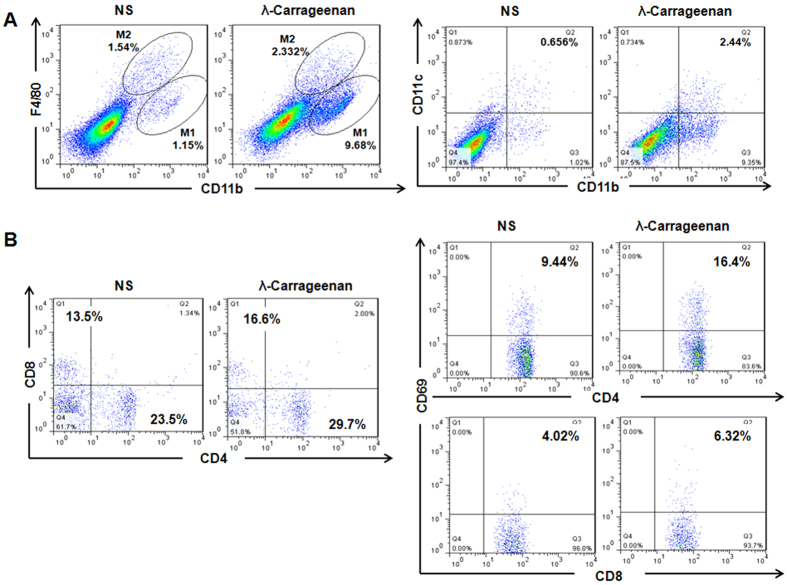
The stimulation of immune response by intratumoral injection of λ-carrageenan in mice. (**A**) Infiltrated macrophages (left) and dendritic cells (right) in B16-F10 tumor tissues after λ-carrageenan treatment were analyzed by flow cytometry. Total numbers of 20000 cells were analyzed. M1 and M2 macrophages were illustrated and the numbers indicate the percentage of the cells in total cells. (**B**) Splenic lymphocytes were analyzed by flow cytometry after λ-carrageenan treatment. Total numbers of 20000 cells were collected. Numbers illustrated indicate the percentage of the cells in total cells. NS is for normal saline group as control. Independent experiments were repeated three times with similar results.

As λ-carrageenan showed low cytotoxicity *in vitro* after incubation with tumor cells, we next investigated how λ-carrageenan affected tumor microenvironment *in vivo* after intratumoral injection in mice. Tumor microenvironment contains many distinct cell types and the immune cells play crucial roles, such as F4/80^+^ macrophages and CD11c^+^ dendritic cells, which are important in initiation of immune response and in antigen-presenting process, respectively^28^. We have evaluated the immune cells in tumor microenvironment after the intratumoral injection of λ-carrageenan by flow cytometry. As shown in Fig. 3A (left), the proportion of F4/80^low^ macrophages was dramatically increased by 10 times after λ-carrageenan treatment while compared with control group. In addition, CD11c^+^ DCs were also increased from 0.6% to 2.4% in tumor tissues (Fig. 3A, right). These results indicated that a large number of F4/80^low^ macrophages and DCs infiltrated into tumor tissue in respond to λ-carrageenan injection.

Furthermore, T lymphocytes were isolated from mice spleen, stained with antibodies to CD4 and CD8 and evaluated by flow cytometry. The results showed that λ-carrageenan treatment enhanced the spleen lymphocyte proliferation. In addition, the spleen index (spleen weight/ body weight) of the mice in λ-carrageenan group was higher than that in NS group, increased from 0.016 to 0.021 (Date not shown). As shown in Fig. 3B (left), we found that CD4^+^ CD8^+^ T lymphocytes have both increased, from 23.5% to 29.7% and 13.5% to 16.6% respectively. Moreover, the activation of splenic lymphocytes was also assessed by staining of marker CD69 in Fig. 3B (right), which is a lymphocyte activation antigen and whose rapid expression makes it possible for the early detection of T-cell activation^29^. These results indicated that λ-carrageenan treatment enhanced both the proliferation and activation of CD4^+^ CD8^+^ T cells simultaneously.

### λ-carrageenan treatment increases the expression of IL17A and TNF-α

**Figure 4:**
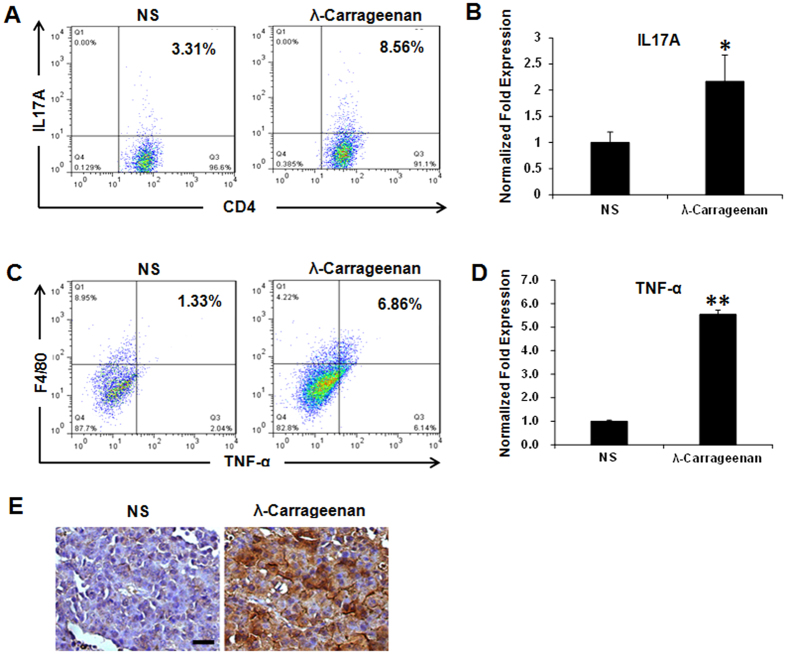
λ-carrageenan treatment improves the expression of IL17A and TNF-α *in vivo*. **(A)** The increase of IL17A-positice CD4-positive lymphocytes in spleen after λ-carrageenan treatment in B16-F10-bearing mice. Total numbers of 100000 cells were collected and CD4-positive splenic lymphocytes were gated. Numbers illustrated indicate the percentage of the cells. (**B**) The secretion of IL17A in splenic cells was detected by qRT-PCR. Results were normalized to GAPDH. (**C**) The increased secretion of TNF-α by F4/80-positive macrophages in B16-F10 tumors after λ-carrageenan treatment. Total numbers of 100000 cells were collected. CD11b-positive cells in tumor were gated. Numbers illustrated indicate the percentage of the cells. (**D**) The level of TNF-α in tumor homogenates was detected by qRT-PCR. Results were normalized to GAPDH. (**E**) The immunohistochemical staining of TNF-α in B16-F10 tumor section after λ-carrageenan treatment. Scale bar, 50 μm. NS is for normal saline group. For intracellular cytokine staining in flow cytometry, cells were fixed/permeabilized and stained with antibody overnight. Independent experiments were repeated three times with similar results. Results were expressed as average ± SD (*n* = 3, ANOVA, **p < 0.01).

We also examined the proportion of T helper cell 17 (Th17) in mice spleen with staining of intracellular IL17A by flow cytometry and qRT-PCR. Th17 recruits and activates neutrophils and plays an important role in immune responses to fungi and extracellular pathogens^30^. We found that intratumoral injection of λ-carrageenan led to an increase in Th17 cells and the increased secretion of IL17A in spleen lymphocytes (Fig. 4A), which might contribute to the stimulation of immune response in tumor-bearing mice after λ-carrageenan treatment.

TNF-α is considered to be involved in immunomodulation and systemic inflammation^31^. Both activated macrophages and most Th17 cells can produce high levels of TNF-α^32^,^33^. Thus we further investigated whether the expression of TNF-α was increased in tumor tissue after λ-carrageenan treatment. The flow cytometry analysis of tumor tissues suggested that TNF-α secreted by CD11b^+^F4/80^+^ macrophages increased five times as compared with the control group (Fig. 4C). Also, the TNF-α mRNA expression level in B16-F10 tumors increased 5.5 times after λ-carrageenan treatment while compared with NS group (Fig. 4D). Furthermore, immunostaining of TNF-α in tumor tissues also suggested the increase of TNF-α after λ-carrageenan treatment as shown in Fig. 4E.

### λ-carrageenan as an adjuvant to enhance OVA-based vaccine potency

As λ-carrageenan significantly stimulated tumor immune response and increased the influx of F4/80^low^ macrophages and DCs which are very important cells in the antigen presenting process, we hypothesized that the λ-carrageenan might be used as a potential adjuvant in cancer immunotherapy. Here we established a murine lymphoma model by subcutaneous injection of ovalbumin (OVA)-expressing E.G7 cells in mice. E.G7-OVA cell line was originally derived from the murine thymoma line, EL-4, by transfection with a neomycin-selectable vector expressing full-length chicken ovalbumin^34^. Ovalbumin, abbreviated as OVA is the main protein found in egg white and is an important model protein in several different areas of research, which is usually chosen as a model antigen to study the efficiency of tumor vaccine and its adjuvant^35^,^36^,^37^.

**Figure 5:**
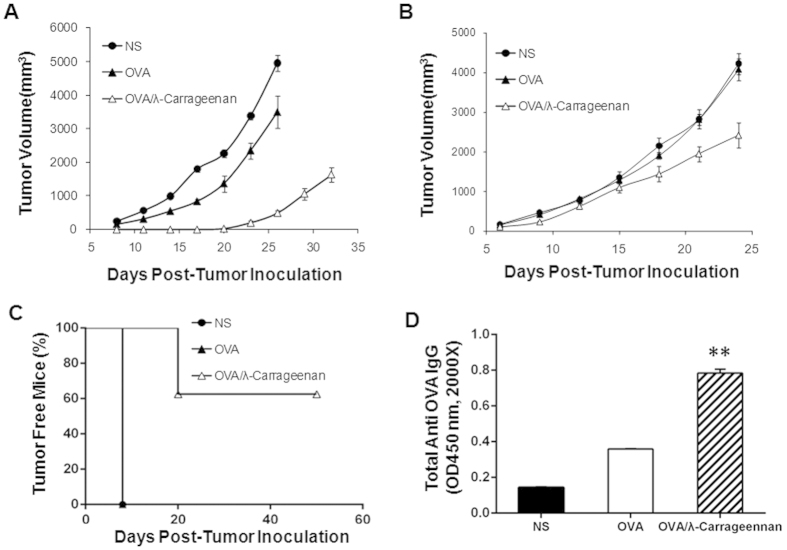
Adjuvant effect of λ-carrageenan in tumor vaccine. (**A**) The preventative vaccines with or without λ-carrageenan were used for E.G7-OVA tumor treatment. Mice were immunized for three times and challenged with E.G7-OVA subcutaneously. Tumor volumes were recorded and were expressed as average ± SEM (*n* = 8). (**B**) The therapeutic vaccines with or without λ-carrageenan were used for E.G7-OVA tumor treatment. Mice were inoculated with E.G7-OVA subcutaneously, then, mice were treated with 3 vaccinations. Tumor volumes were recorded and were expressed as average ± SEM (*n* = 8). (**C**) Percentage of tumor-free mice after immunization with OVA alone or in combination with λ-carrageenan (*n* = 8). (**D**) The detection of anti-OVA antibody in mouse serum by ELISA. Values represent the absorbance of 2000-fold diluted serum. Data represent the mean ± SD (*n* = 3). **p < 0.01 compared to OVA group. NS is for normal saline group.

We have evaluated the efficiency of λ-carrageenan as an adjuvant in the use of both preventative vaccine and therapeutic vaccine in mice. In the evaluation of cancer preventative vaccine, mice were vaccinated three times with OVA with or without λ-carrageenan and then challenged with E.G7-OVA cells (3 × 10^6^ cell/mice). The volumes of tumors were recorded. The results showed that the vaccines with λ-carrageenan added as an adjuvant significantly inhibited the tumor growth while compared with normal saline and OVA group (Fig. 5A). When it comes to the therapeutic vaccine, injection of OVA showed no therapeutic effect while compared with the control, in contrast to that, injection of OVA/λ-carrageenan inhibited tumor growth to some extent while compared with other groups (Fig. 5B). After the mice were immunized with preventative vaccine with λ-carrageenan, six out of ten mice were tumor-free for more than 40 days after tumor inoculation, while all the mice in NS group and OVA group bared tumors (Fig. 5C). One week after final immunization in mice, mice serum were collected and tested by ELISA for evaluation of total anti-OVA IgG. As shown in Fig. 5D, the antibodies generated in mice immunized with OVA/λ-carrageenan were significantly increased while compared with other two groups. The absorbance rates of samples in OVA and OVA/λ-carrageenan group were 0.35 and 0.78 respectively, which represented a four times increase of the antibody titer in OVA/λ-carrageenan group while compared to OVA group (from 2000 to 8000).

### Tumor Histopathology study and toxicity evaluation

**Figure 6:**
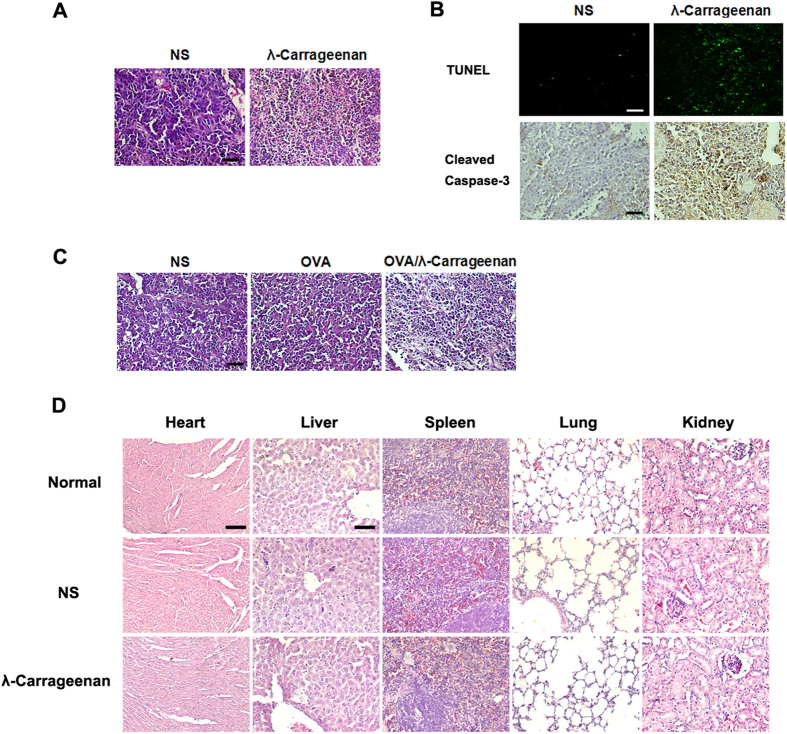
Histopathology of tumors and vital organs in mice after λ-carrageenan treatment. (**A**) H&E staining of B16-F10 tumors after intratumoral injection of λ-carrageenan. Scale bar, 100 μm. (**B**) TUNEL assay and immunohistochemical staining of cleaved caspase-3 in B16-F10 tumor after intratumoral injection of λ-carrageenan. Scale bar, 100 μm (**C**) H&E staining of E.G7-OVA tumors after therapeutic vaccination with OVA with or without λ-carrageenan. Scale bar, 100 μm. (**D**) H&E staining of mouse vital organs after subcutaneous injection of λ-carrageenan in mice. Scale bar, 200 μm for heart and 100 μm for others.

In the present study, hematoxylin and eosin stainning were performed to study the tumor morphology after λ-carrageenan treatment. As shown in Fig. 6A, B16-F10 tumor tissues from the λ-carrageenan intratumoral injection group had some area of cell necrosis and significant increase of immune cell infiltration. However, tumor sections from normal saline treated mice remained normal morphology. We have also performed TUNEL assay and cleaved caspase-3 staining for evaluation of cell death in tumor sections. As shown in Fig. 6B, the positive cells for TUNEL and cleaved caspase-3 were both significantly increased after intratumoral injection of λ-carrageenan, which might contribute to the significant antitumor effect of λ-carrageenan. The tumor sections of E.G7-OVA bearing mice treated with or without therapeutic vaccines were also presented (Fig. 6C). Mice immunized with OVA/λ-carrageenan also showed increase in cell death and more immune cell infiltration in tumors.

Moreover, to examine potential toxicity of λ-carrageenan, vital organs in λ-carrageenan treated mice (heart, liver, spleen, lung and kidney) were collected and sections were stained with H&E for histopathological study. As shown in Fig. 6D, no significant pathologic changes were found in λ-carrageenan treated mice. In addition, no obvious toxicities were observed in the mice as determined by appearance, body weight, fecal and urinary excretion.

## Discussion

λ-carrageenan is a mucopolysaccharide separated from the cell walls of marine red algae and is widely used in the studies concerned with inflammation^23^. However, how the proinflammatory property of λ-carrageenan affects tumor microenvironment is largely unknown. In the present study, we have investigated how λ-carrageenan affected tumor growth via intratumoral injection and the potential of λ-carrageenan used as the adjuvant in cancer vaccine. We found that the intratumoral injection of λ-carrageenan significantly inhibited tumor growth through the activation of tumor immune response, such as increasing the influx of antigen-presenting cells. Also, the addition of λ-carrageenan as an adjuvant could notably increase the efficiency of a tumor vaccine. These suggestions are supported by the findings in our study, namely, the intratumoral injection of λ-carrageenan significantly inhibited the tumor growth in murine B16-F10 melanoma model and murine 4T1 mammary tumor model. The addition of λ-carrageenan to cells *in vitro* showed little cytotoxicity to tumor cells even at a higher concentration as detected by trypan blue exclusion assay and MTT assay. λ-carrageenan injection dramatically increased the proportion of M1 macrophages and DCs in tumor microenvironment and also increased the activated lymphocytes and Th17 cells in spleens in treated mice as detected by flow cytometry and qRT-PCR. The increased secretion of TNF-α was detected in the tumor tissues by flow cytometry, qRT-PCR and immunohistochemistry. Interestingly, we also found that the application of λ-carrageenan as an adjuvant in the preventative tumor vaccine notably reduced the percentages of tumor-bearing mice from 100% to 40% as compared with OVA group and NS group. The therapeutic vaccine also inhibited tumor growth to some extent with λ-carrageenan added as an adjuvant. The tumor sections were made and stained with H&E, TUNEL and cleaved caspase-3 and increased cell apoptosis was observed. The H&E staining of vital organs from the λ-carrageenan treated mice showed no abnormalities while compared with controls. Based on these results, we draw a conclusion that λ-carrageenan is a potent anti-tumor agent while administered intratumoraly or used as an adjuvant in tumor vaccine, which inhibited tumor growth through stimulating immune response while exhibiting no toxicities to other vital organs.

The suppressive tumor microenvironment is crucial for the survival, proliferation, and migration of cancer cells and has received growing attention recently^38^. Considerable efforts have been devoted in developing the treatment to overcome the immunosuppressive barriers in cancer therapy^39^,^40^,^41^. As one category of the stromal cells in tumor microenvironment, tumor associated macrophages (TAMs) help to keep balance in favor of tumor progression by suppressing tumor immune response^42^, such as reducing the cytotoxic CD8^+^ T-lymphocyte activity^43^. Depending on the environment, activated macrophages can be separated into two distinct phenotypes: M1 (classical activated, normally inhibit tumor growth) stained as F4/80^low^ cells and M2 (alternative activated, pro-tumoral) stained as F4/80^high^ cells^44^. Tumor resident macrophages consistently present a highly immunosuppressive M2 profile whereas the newly infiltrated macrophages are generally immunostimulating and cytotoxic to tumor cells which belong to M1 macrophages^44^,^45^,^46^,^47^. In this study, the intratumoral injection of λ-carrageenan significantly induced the infiltration of M1 macrophages (detected as CD11b^+^F4/80^low^ in flow cytometry) in tumor tissues and increased the production of proinflammatory cytokines which helped to improve the tumor immune response together with the infiltrated DCs and activated lymphocytes.

TNF-α is one of cell factors that mainly secreted by activated M1 macrophages, which could recognize the receptors on tumor cell membranes and kill tumor cells specifically^32^,^33^. In the present study, results of flow cytometry suggested that the production of TNF-α was increased and secreted from the infiltrated macrophages in tumor microenvironment in response to λ-carrageenan stimulation. The increased infiltration of F4/80^low^ macrophages and the subsequent secretion of TNF-α in tumor tissues might contribute to the antitumor effect of λ-carrageenan.

Moreover, intratumoral injection of λ-carrageenan led to an increase of proinflammatory IL17A (Fig. 4A), which was secreted by a subset of T helper cells, Th17 cells. Most Th17 cells can produce high levels of effector cytokines such as TNF-α and can promote antitumor immune responses by inducing the recruitment of proinflammatory immune effector cells^48^,^49^. This also supports our conclusion that intratumoral injectioin of λ-carrageenan could enhance tumor immune response. Besides, Malley *et al.*^50^ have reported the role of IL17A in the immune response during vaccine application against pneumococci. Increase of Th17 cells induced by addition of adjuvants might significantly help to improve the efficiency of a vaccine. In this study, we also used λ-carrageenan as an adjuvant in both preventative vaccine and therapeutic vaccine for cancer treatment. λ-carrageenan acted as an effective adjuvant for OVA-based vaccine in OVA-expressing E.G7 lymphoma model. Also, λ-carrageenan enhanced the production of the anti-OVA antibody in mice after immunization. This indicated that mice immunized with λ-carrageenan as the adjuvant showed strong humoral responses, which might be mediated by antibodies produced by B lymphocytes.

In summary, here we have provided insights into the role of λ-carrageenan used for cancer therapy both as antitumor agent and vaccine adjuvant. λ-carrageenan exhibited considerable antitumor effect via intratumoral injection and significantly improved the tumor immune response with increased infiltrates of immunostimulating cells and increased production of proinflammatory cytokines. While used as vaccine adjuvant, λ-carrageenan notably increased the efficiency of both preventative and therapeutic cancer vaccines. In addition, injection of λ-carrageenan showed no toxicities to vital organs in treated mice. Thus, we conclude that λ-carrageenan is a potent antitumor agent and efficient adjuvant which might be further used in cancer treatment to enhance tumor immune response.

## Methods

### Animals and cell lines

Female C57BL/6 or BALB/c mice purchased from Vital River, Peking, China, were placed in a specific pathogen-free (SPF) environment with a consistent room temperature and humidity. Animal experiments were performed according to the guidelines of the Animal Care and Use Committee of Sichuan University (Chengdu, Sichuan, China) and approved by animal association.

The murine melanoma cell line B16-F10, murine mammary tumor cell line 4T1 and E.G7-OVA (an EL4 cell line that is transfected by electroporation with the cDNA of OVA to allow endogenous production of OVA with an H-2K^b^-restricted CTL epitope) were obtained from the American Type Culture Collection (ATCC). Both cells were grown and maintained in RPMI medium 1640 supplemented with 10% FBS and E.G7-OVA were maintained in medium supplemented with another 400 μg/mL G418. Cells were maintained in a 37 ^o^C incubator with 5% humidified CO_2_ atmosphere.

### Tumor models and λ-carrageenan treatment

λ-Carrageenan was purchased from Sigma-Aldrich (cat.22049). B16-F10 cells (5×10^5 ^cell/mice) were injected subcutaneously in the right dorsal flank of C57BL/6 mice. 4T1 cells (1×10^6^ cell/mice) were injected subcutaneously into the right dorsal flank or injected subcutaneously into mice fat pad of the mammary gland of BALB/c mice to establish the tumor model. The administration started after the tumors reached the average volume of 30–40 mm^3^. λ-carrageenan (1%) dissolved in saline was injected intratumoraly in a volume of 100 μL every two days and the negative control were injected with 100 μL of normal saline (NS). Adriamycin (Melone Pharmaceutical Co., Ltd, DaLian, China) was used as an anti-tumor agent for positive control. The mice were treated with adriamycin (5 mg/kg) via intraperitoneal injection every three days. Tumor growth was evaluated by measurement of tumor diameters using a caliper every 3 days and tumor volume was calculated as (long diameter) × (short diameter)^2^ × 0.52. The mice were sacrificed two days after final administration and tumors and organs were harvested, weighed and fixed in 4% paraformaldehyde for histochemistry; another part of tumors were stored in liquid nitrogen for further use. Inhibition ratio was calculated by following formula: Inhibition ratio (%) = [(A−B)/A]×100, where A represents the average tumor weight of the negative control, and B represents that of the λ-carrageenan treated group.

### Cell viability assay *in vitro*

Cell viability was measured using trypan-blue exclusion assay and MTT (Sigma Aldrich) assay. B16-F10 and 4T1 cells were plated into 96-multi-well plates and cultured overnight to achieve cell adhesion, respectively. Then cells were treated with different concentrations (0.25, 0.5, 1, 2.5 mg/mL) of λ-carrageenan. After 24 and 48 h of treatment, we replaced the fluid with new culture media containing MTT (0.5 mg/mL) and incubated for 4h until purple precipitate was visible. MTT was removed and 150 μL DMSO was added to dissolve the precipitate. Absorbance was measured at 570 nm using a microplate reader. Each concentration was replicated 6 wells. The blank group and control group were set up simultaneously. For Trypan blue exclusion assay, the cells were treated as MTT assay. After λ-carrageenan treatment, the status of cells was first evaluated by microscopy. Then cells were harvested and 10 μl of cell suspension was mixed with 10 μl of 0.4% Trypan blue solution (Beyotime Institute of Biotechnology, Shanghai, China) for 3 min, and both unstained (viable) and stained (dead) cells were counted by the Countstar Automated Cell Counter.

### Flow cytometry analysis

After the treatment of λ-carrageenan, immune cells prepared from spleen and tumors were stained with PE or PerCP-CD4, FITC-CD8, PE-CD69, FITC or PerCP-CD11b, PE-F4/80, PE-CD11c, FITC-IL17A, FITC-TNF-α. All the fluorophore conjugated antibodies and isotype-matched mAbs were purchased from BD Pharmingen. Tumors were scissored into small pieces, and dissociated using 1 mg/mL collagenaseI in serum free RPMI 1640 medium for 2 hours. Resulting cell suspensions were centrifuged, washed in PBS and passed through a BD Falcon™ 70-μm nylon cell strainer to remove clumps of cells and debris. For cell surface staining, cells were stained with antibodies on ice for 30 min in the dark; for intracellular cytokine staining, cells were fixed/permeabilized with paraformaldehyde and Triton-X100 and then stained with intracellular antibodies overnight. Analyses were carried out on a FACS Calibur flow cytometer (BD Biosciences) and data were analyzed using FlowJo software.

### Quantitative PCR Analysis

Reverse transcription polymerase chain reaction (RT-PCR) was employed to measure IL17A and tumor necrosis factor-α (TNF-α) mRNA transcription levels in spleen lymphocytes and tumor tissues respectively. Spleen lymphocytes were enriched by lymphocyte separation medium (Dakewe Bioengineering Co., Beijing, China) according to the manufacturer’s instructions. Tumor samples were ground in liquid nitrogen. Total RNA from spleen lymphocytes or tumor homogenates were isolated using RNAsimple Total RNA Kit (TIANGEN, Peking, China). 1μg isolated RNA of each experimental groups was used to synthesize cDNA using Takara kit (Takara, Dalian, China) following the manufactures’ instructions. The primers used were as follows: IL17A, 5’-TTT AAC TCC CTT GGC GCA AAA-3’ (forward) and 5’-TTT AAC TCC CTT GGC GCA AAA -3’ (Reverse); TNF-α, 5’-TCT TCT CAT TCC TGC TTG TGG-3’ (forward) and 5’-GGT CTG GGC CAT AGA ACT GA-3’ (Reverse); GAPDH, 5’-ACC CAG AAG ACT GTG GAT GG-3’ (forward) and 5’-CAC ATT GGG GGT AGG AAC AC-3’ (Reverse). Real-time PCR was performed with SsoAdvanced SYBR Green supermix (BIO-RAD, USA) using a Two-step PCR reaction procedure. Expression of the IL17A and TNF-α gene was normalized to the expression of GAPDH. Data were analyzed by the 2^−ΔΔCt^ method.

### Antitumor immunity experiment of OVA and λ-carrageenan

For the preventative tumor vaccine experiment, the left dorsal flank of C57BL/6 mice (*n* = 8/group) were vaccinated s.c. 3 times (at 0, 2 and 3 weeks) with 5 μg OVA protein along or OVA protein plus 100 μg λ-carrageenan as adjuvant at a total volume of 100 μL NS. One week after the last immunization, mice were challenged with E.G7-OVA cells. The tumor cells (3 × 10^6^) were injected s.c. into the right dorsal flank in mice. The subcutaneous tumor volume was measured every three days. The tumor formation rate of mice was observed every day.

For the therapeutic antitumor vaccine experiment, EG.7-OVA cells were s.c. inoculated into the right dorsal area of C57BL/6 mice. When the tumor mass became palpable (about 3 mm in length), the tumor-bearing mice were treated by 3 vaccinations with one week interval in the left dorsal area. The subcutaneous tumor volume was measured as mentioned above.

### Anti-OVA IgG Detection

Blood was collected 7 days after the last immunization of mice with OVA. Serum were tested by ELISA for total anti-OVA IgG. NUNC Maxisorp plates were coated overnight at 4 °C with 100 μl of OVA (10 μg/mL) in coating buffer (pH9.6). The plates were then washed three times with PBST (PBS containing 0.01% Tween 20) and blocked for 1h at 37 ^o^C with 100 μL/well ELISA buffer (5% skimmed milk solution in PBST). The plates were then washed three times with PBST. 100 μL Mouse serum was serially diluted with ELISA buffer, added into the well and incubated for 2 h at 37 °C. After washing the plate with PBST for five times, horseradish peroxidase (HRP)-conjugated goat anti-mouse IgG (diluted 1:10,000 in ELISA buffer) was added (100 μl/well) and incubated for 1 h at 37 °C. The plates were then washed for five times with PBST. Finally, the samples were developed with 100 μL TMB substrate for 10 min at room temperature and then stopped with 50 μL of 1M H_2_SO_4_. Absorbance was measured at 450 nm using a microplate reader.

### Histochemistry and Immunohistochemistry

To evaluate potential toxicity of the treatment, B16-F10 tumor and mouse organs (heart, liver, spleen, lung and kidney) were harvested and fixed with buffered 4% paraformaldehyde for 72 h and embedded in paraffin wax. 4 μm sections were stained with hematoxylin and eosin (H&E). Histological sections of E.G7-OVA tumors in the therapeutic vaccine experiment were also stained with H&E.

For immunohistochemistry, tumor sections were deparaffinized, rehydrated in graded series of alcohol and antigen retrieval was performed in citrate buffer (pH 6.0) for 3 minutes in an autoclave. Endogenous peroxidase activity was inhibited by incubation with 3% H_2_O_2_ for 15 min in the dark. After blocking nonspecific reactivity with normal goat serum for 40 min at 37 °C, samples were incubated overnight at 4 °C with rabbit anti-mouse TNF-α antibody (Abcam, 1:100) or anti-mouse cleaved caspase-3 antibody (Cell signaling technology, 1:300), followed by the incubation with biotinylated goat anti-rabbit secondary antibody at 37 °C for 40 min and streptavidin-biotin complex at 37 °C for another 40 min. The immunoreaction was developed using diaminobenzidine peroxide solution. Cell nuclei were gently counterstained with hematoxylin. Images were obtained with a Leica DM 2500 microscope.

### TUNEL Assay

To examine the cell death in B16-F10 tumors after intratumoral injection of λ-carrageenan, paraffin sections of tumor tissue specimens were stained with terminal deoxynucleotidyl transferase-mediated deoxyuridine triphosphate-biotin nick-end labeling (TUNEL) using a commercially available TUNEL kit (Promega, Madison, WI, U.S.) according to the manufacturer’s instructions. Samples were observed under a DM 2500 fluorescence microscope (Leica Microsystems CMS GmbH, Wetzlar, Germany).

### Statistical Analysis

All data expressed as mean ± SD or mean ± SEM are representative of at least three independent experiments. Data were statistically evaluated using one-way analysis of variance (ANOVA) test. The values were considered statistically significant when *p* < 0.05 (signified by*) and *p* < 0.01(signified by**).

### Ethics statement

The methods were carried out in accordance with the approved guidelines. The Animal Care and Use Committee of Sichuan University (Chengdu, Sichuan, China) approved all the animal experiments.

## Additional Information

**How to cite this article**: Luo, M. *et al.* Antitumor and Adjuvant Activity of λ-carrageenan by Stimulating Immune Response in Cancer Immunotherapy. *Sci. Rep.* **5**, 11062; doi: 10.1038/srep11062 (2015).
